# Clinical report of microsurgical treatment of Kohler's disease

**DOI:** 10.1038/s41598-024-57088-w

**Published:** 2024-03-15

**Authors:** Yantao Pei, Lei Zhu, Qingjia Xu, Juntao Wang, Yuliang Sun, Gang Wang

**Affiliations:** Department of Orthopedics, Department of Hand Surgery, Foot and Ankle Surgery, Qilu Hospital of Shandong University, Jinan, 250012 China

**Keywords:** Kohler's disease, Avascular navicular necrosis, Vascular bundle implantation, Surgical treatment, Microsurgery, Diseases, Medical research, Signs and symptoms

## Abstract

The conservative treatment for Kohler's disease will take several months, but some patients still have flatfoot and persistent pain. From October 2013 to July 2015, 3 children with Kohler's disease underwent navicular decompression and micro-circulation reconstruction surgery in our hospital. All the patients have received conservative treatment for more than 3 months and the effect was poor. X-ray showed the bone density of navicular increased significantly. All patients were followed up over 1 year. The 3 patients recovered well. VAS score decreased from 7.0 to 2.6 at 1 month after the operation. The pain symptom disappeared completely on 3 months after surgery. The density of navicular bone recovered to normal. Navicular decompression and micro-circulation reconstruction surgery may quickly improve the ischemic status of navicular bone, alleviate pain symptom and enable patients to resume normal activity as soon as possible.

## Introduction

Avascular necrosis of the navicular bone in children, also known as Kohler's disease, was first reported by Dr. Kohler in 1908^1,2^. Kohler's disease is one of the rare diseases of foot and ankle, which is prone to be misdiagnosed and missed^3,4^.

This disease is characterized with abnormal endochondral ossification and osteocytes necrosis, which is mainly caused by blood circulation disorder of the navicular bone due to trauma, growth restriction, or other disease^3^. Symptoms of Kohler's disease include regional swelling, pain, and claudication, the severity varies from walk-on lateral side of the foot to inability to walk. The typical radiological manifestations are navicular bone sclerosis, fragmentation, and flatten, sometimes with the medial arch collapse^5^.

Kohler's disease is self limiting and patients may recover through standardized conservative treatment^1,6,7^. However, the symptoms may persist several months and even years^8^, which seriously affect the life quality of patients. Conservative treatment include controlled ankle motion boot, or use of crutches while weight bearing was tolerated^1^. Short leg cast is for patient with more severe symptoms and have difficulty weight bearing, but it should not be used exceed 6 weeks to avoid ankle stiffness. Non steroidal anti-inflammatory drugs may need to reduce pain symptom. Although Kohler's disease may recover following appropriate conservative treatment, patients with Kohler's disease are usually in the rapid growth ages and challenging to cooperate with long-term cast protection^9^. In this study, we treat Kohler's disease with navicular decompression and vascular implantation to reconstruct the blood circulation, and report the results.

## Materials and methods

### Patients

From October 2013 to July 2015, 3 boys with Kohler's disease underwent surgical treatment of navicular decompression and vascular implantation. The 3 patients were 5.0, 4.5 and 5.3 years old with an average age of 4.9 years old.

All the patients have a history of claudication due to pain in the medial part of right midfoot for 4–6 months, the pain aggravated gradually and patients were hardly able to walk. Conservative treatment like controlled ankle motion boot or short leg cast were used in our clinic for over 1 month with poor result. The total time of conservative treatment was more than 3 months, and the patients cannot continue to tolerate cast or external brace and conservative therapy has failed. The patients were otherwise healthy and denied any family history of limb deformity.

### Pre- and post-operative assessment

All the patients underwent physical examination and X-ray before and after the operation. 2 patients had MRI examination before the operation.

### Surgical procedure

The patients were operated in supinate position under general anesthesia with a tourniquet on the thigh. Anterior-medial approach was used, and the incision was from the tip of the medial malleolus to the distal of the cuneonavicular joint. The extensor hallucis longus tendon and extensor digitorum longus tendon were retracted, and the dorsalis pedis artery and the medial tarsal artery were exposed at the talonavicular joint level.

**Figure 1:**
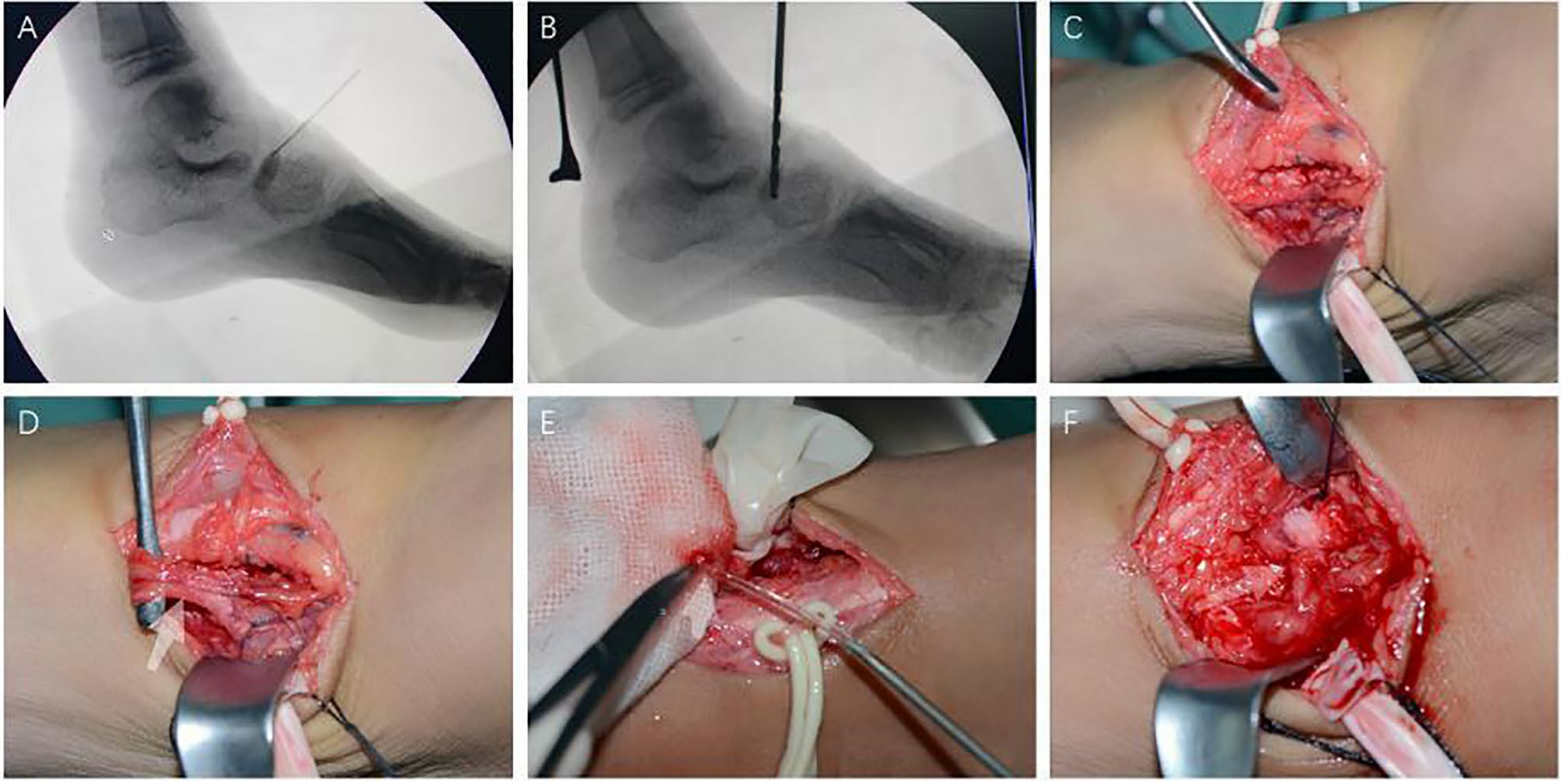
The surgical procedure of vascular bundle implantation to treat Kohler's disease. (**A**) Located the navicular under fluoroscopy; (**B**) Drill under fluoroscopy; (**C**) and (**D**) Exposed the dorsal pedal artery and the medial tarsal artery at the talonavicular joint level; cut the medial tarsal artery and accompany veins, (**E**) Showed bleeding of the proximal end(Arrow); (**F**) Vascular bundle implantation into the drilled hole of the navicular.(Arrow).

The medial tarsal artery was dissociated about 3 cm. The navicular was exposed and a 2–3 cm deep hole was drilled on the dorsal side with a 3.0 mm drill for decompression. Care was exercised to not drill through the contralateral cortex to prevent excessive damage and bleeding. The medial tarsal artery was cut off, the distal part of the vessel was ligated and the proximal end was kept open, and blood flowing from the artery and accompanying veins could be seen. The opened proximal end was inserted into the drilled hole of the navicular. The medial tarsal artery was fixed on the periosteum of navicular by absorbable suture. The incision was closed and the foot was fixed in the neutral position with splint. The surgical procedure was shown in Fig. 1.

### Postoperative treatment

The affected feet were fixed with splint for 3 weeks. The patient began weight-bearing stand training at 4 weeks after operation, walk slowly and gradually return to normal walking.

### Follow-up

The patients were followed up once a month after the operation to check whether their feet have swelling, pain or infection. X-ray check were taken every month within 3 months after the operation. After 6 months, the foot condition was checked every 3 months, the epiphyseal closure, foot arch development and local symptoms were recorded.

### Ethical approval and Informed consent

This study was approved by the Ethics Committee of Qilu Hospital of Shandong University. All procedures performed in this study involving human participants followed the relevant guidelines and regulations of the Declaration of Helsinki. All patients in our study were anonymous. Informed consent was obtained from all individual participants included in the study.

## Results

**Figure 2:**
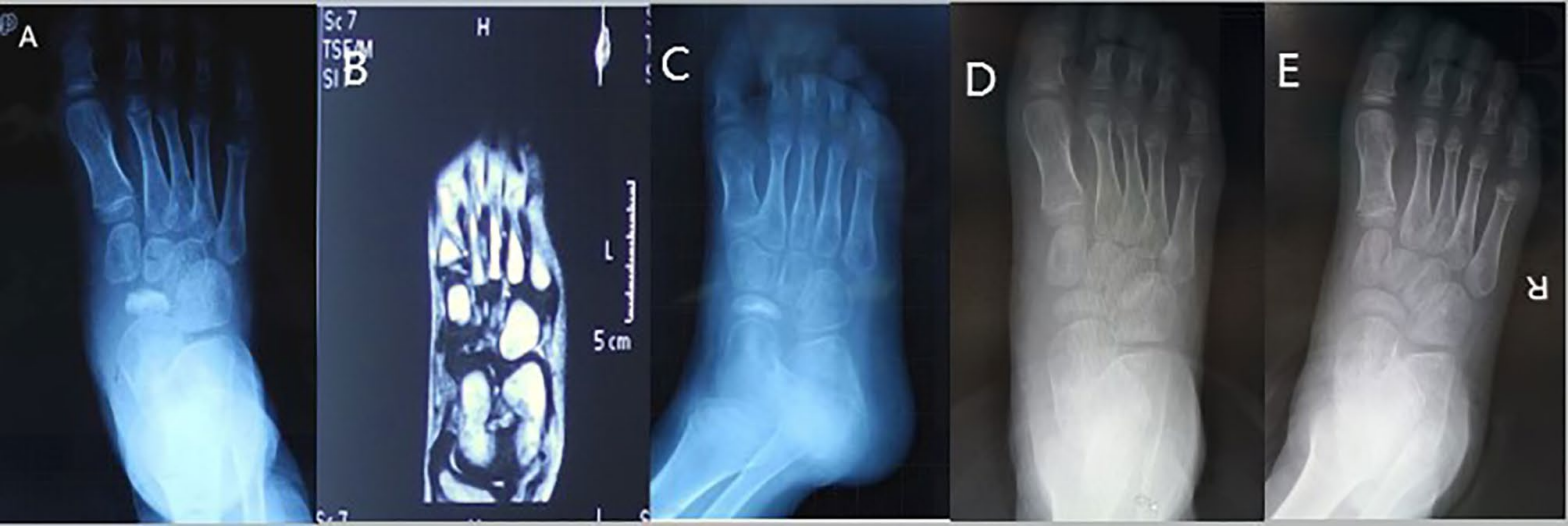
Case 1 radiography changes of the navicular after vascular bundle implantation. (**A**) Pre-operation X ray showed navicular bone sclerosis; (**B**) Pre-operation MRI showed ischemic status of the navicular bone; (**C**) 1 month post-operation X ray showed radiography improvement of the sclerotic navicular bone; (**D**) 3 months post-operation X ray; E, 6 months post -operation X ray.

**Figure 3:**
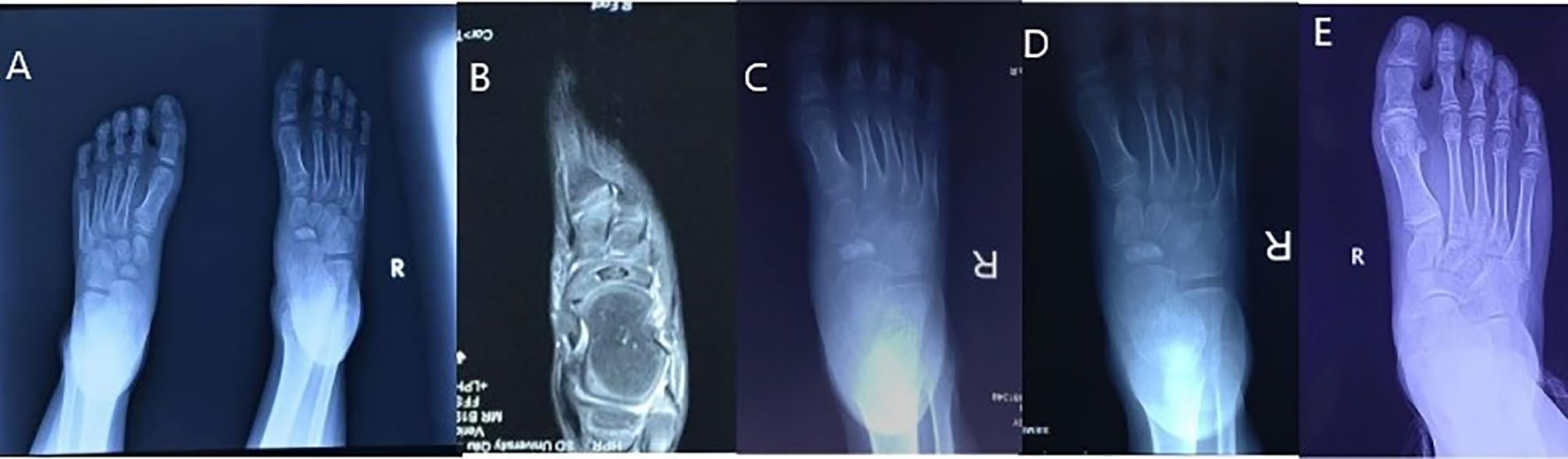
Case 2. (**A**) Pre-operation X ray showed the right navicular bone sclerosis,compared with the left foot; (**B**) Pre-operation MRI showed ischemic status of the navicular bone; (**C**) 1 month post-operation X ray showed radiography improvement of the sclerotic navicular bone; (**D**) 2 months post-operation X ray; (**E**) 7 years post-operation X ray.

The 3 patients recovered well after the navicular decompression and micro-circulation reconstruction surgery. VAS score decreased from over 7 preoperative to 2.6 at 1 month after the operation. There was no pain and tenderness on their foot at 3 months postoperative. All patients had noticeable radiography improvement at 2 months, and radiographic recovery at 3 months. All patients resume normal walking within 2 months after surgery. The density of navicular reduced, normal trabecula pattern recovered, the shape of navicular became normal. (Fig. 2) All patients resume normal activities at 3 months after the surgery. Until the last follow-up, no recurrence was recorded, and the navicular developed naturally, same as the contra lateral (Fig. 3).

## Discussion

Kohler's disease is believed to be a self-limited disease, but conservative treatment usually need several months, and sometimes may lead to failure. Conservative treatment include controlled ankle motion boot, crutches, short leg cast and drugs. Conservative treatment is the first choice of patients and doctors, but it is very difficult for young patients to cooperate with long-term external fixation. We need to find ways to quickly and thoroughly solve the problems. The vascularized bone transfer in the form of the medial femoral condyle free flap was a valuable method for navicular avascular necrosis^10^, but need microsurgical technique and vessel anastomosis. Core decompression have demonstrated favorable outcomes in treatment of Kienböck disease and scaphoid necrosis^11^. But these method had never been used in treat the Kohler's disease. We performed navicular decompression and micro-circulation reconstruction on 3 patients, and the results showed that the course of treatment was short, and the curative effect was definitive. But surgery also carries the risk of foot scars and tendon adhesion.

The etiology of Kohler's disease remains unclear. As the navicular on the top of the medial arch, and under great stress among the talus and cuneiform^10,12^. Moreover, the ossification center of the navicular, which is the latest, may be affected by the stress and lead to the avascular necrosis of the navicular bone^13,14^. Drill on the bone may reduce the internal pressure. Core depression had been used in treat the wrist scaphoid nonunion and get good results. The vascular implantation would restore the blood supply, helping the navicular bone recover from avascular necrosis.

Reconstruction of micro-circulation with vascular implantation has long been used in treating bone ischemic necrosis^15^, but never been used in the treatment of Kohler’s disease. Compared with free vascularized bone transfer, vascular transplantation is a more direct and comfortable operation. Besides oxygen and nutrition, the vascular implantation also brings growth factors and fibroblasts or other cells, as the foundation for bone reconstruction^16^.

We also need to note that despite surgery, there also could be a spontaneous effect of improvement related to rest and cast treatment. In our study, the affected feet were fixed with splints for 3 weeks, which was shorter than conservative treatment. More comparative studies were needed in the future to clarify the effectiveness of surgical treatment.

## Conclusions

Navicular decompression and micro-circulation reconstruction by vascular implantation to treat Kohler's disease, the results showed the sclerotic bone quickly recovered and the symptoms alleviated. Though more cases observation are needed, we believe this procedure might be an promising treatment method. The indication for surgery is only patients with severe pain and after failed conservative therapy of several months.

## Data Availability

The datasets used and analysed during the current study available from the corresponding author on reasonable request.

## References

[1] Chan JY, Young JL (2019). Köhler disease: Avascular necrosis in the child. Foot Ankle. Clin..

[2] Stanton BK, Karlin JM, Scurran BL (1992). Köhler's disease. J. Am. Podiatr. Med. Assoc..

[3] AL Hamdani M, Kelly C (2017). Kohler's disease presenting as acute foot injury. Am. J. Emerg. Med..

[4] Shanley J, James DR, Lyttle MD, Andronikou S, Knight DM (2017). Kohler's disease: An unusual cause for a limping child. Arch. Dis. Child..

[5] Mj COX (1958). Kohler's disease. Postgrad. Med. J..

[6] Karr JC (2020). External fixation diastasis management of Kohler's disease in a 14-year-old boy: A case report. J. Am. Podiatr. Med. Assoc..

[7] Shastri N, Olson L, Fowler M (2012). Kohler's disease. West J. Emerg. Med..

[8] Borges JL, Guille JT, Bowen JR (1995). Köhler's bone disease of the tarsal navicular. J. Pediatr. Orthop..

[9] Houlden R (2021). Does immobilisation improve outcomes in children with Köhler's disease?. Arch. Dis. Child..

[10] Haddock NT, Alosh H, Easley ME, Levin LS, Wapner KL (2013). Applications of the medial femoral condyle free flap for foot and ankle reconstruction. Foot Ankle. Int..

[11] Tadisina KK, Pet MA (2022). Osteotomies, core decompression, and denervation for the treatment of Kienböck disease. Hand Clin..

[12] Williams GA, Cowell HR (1981). Köhler's disease of the tarsal navicular. Clin. Orthop. Relat. Res..

[13] Dyson S, Marks D (2003). Foot pain and the elusive diagnosis. Vet. Clin. North Am. Equine. Pract..

[14] Pourlis AF, Antonopoulos J (2014). The ossification of the pelvic girdle and leg skeleton of the quail (*Coturnix coturnix japonica*). Anat. Histol. Embryol..

[15] Divakov MG (1991). Revascularization of avascular spongy bone and head of the femur in transplantation of vascular bundle (An experimental and clinical study). Acta Chir. Plast..

[16] Han D, Li J (2013). Repair of bone defect by using vascular bundle implantation combined with Runx II gene-transfected adipose-derived stem cells and a biodegradable matrix. Cell Tissue Res..

